# A Primer on Imaging

**Published:** 1995

**Authors:** John J. Doria

**Affiliations:** John J. Doria, M.S., is a science editor of Alcohol Health & Research World

**Keywords:** brain, diagnostic imaging, computed x-ray tomography, magnetic resonance imaging, magnetic resonance spectroscopy, single photon emission computed tomography, positron emission tomography, electroencephalography, magnetoencephalography

## Abstract

During the past 30 years, increasingly sophisticated imaging techniques have been developed for examining the structure and function of the living brain. Computed tomography and magnetic resonance imaging can each represent a three-dimensional “slice” of brain, showing more detail than a conventional X ray. Functional imaging techniques permit scientists to detect changes in blood flow and energy metabolism. Such techniques include magnetic resonance spectroscopy, magnetic resonance imaging, single-photon emission computed tomography, and positron emission tomography. In addition, electroencephalography records the spontaneous electrical activity of the brain, and magnetoencephalography measures and displays the magnetic field that surrounds the head in association with the electrical activity.

The skull is uniquely designed to support the brain and protect it from damage. Yet it is precisely this design that shields it from the view of experimenters and clinicians. Conventional skull X rays, available since the early 1900’s, do not provide sufficient contrast to distinguish different parts of the brain. Until recently, the only practical way to visualize the brain was to examine it after the patient had died. During the past 30 years, however, scientists have developed increasingly sophisticated imaging techniques for examining both the structure and function of the living brain and of other organs as well. The following brief review concentrates on imaging techniques applicable to alcohol research.

## Structural Imaging

### Computed Tomography

The first modern development in brain imaging was the introduction in 1973 of computed tomography (CT), a refinement of standard x-ray technology.^1^ CT uses x-ray cameras that rotate around the patient’s head to measure differences in tissue density across thin slices or sections of brain tissue. A series of images are usually taken, representing serial slices or sections, from the base of the brain to the crown. A computer converts data on relative tissue density into images that can be displayed on a monitor or transferred to x-ray film. The ability of CT and other tomographic methods to visualize discrete slices of tissue eliminates confusing shadows from adjacent overlying structures.

**Figure 1:**
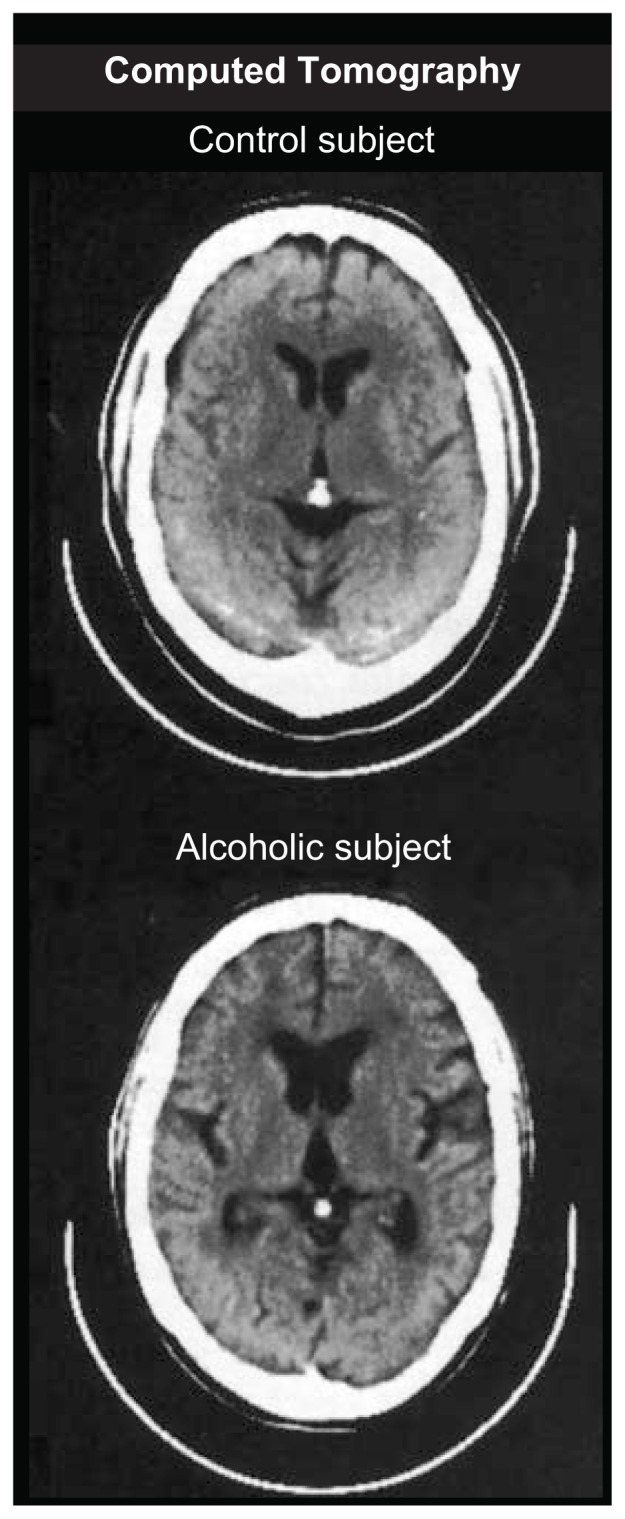
These computed tomography images show loss of brain volume in a 60-year-old alcoholic man compared with a 61-year-old nonalcoholic man. These images depict slices of brain tissue 10 mm thick. Image courtesy of Margaret Rosenbloom.

CT is significantly more sensitive to gradations of tissue density than a conventional skull X ray. With CT images, the most contrast occurs among bone, brain tissue, and cerebrospinal fluid (CSF) (figure 1). Bone appears bright, CSF appears dark, and brain tissue appears somewhat in between. Modern CT scanners produce fine-grained resolution, permitting some differentiation between white and gray matter. CT imaging is not limited to the brain; abdominal organs that are virtually invisible to standard X rays—such as the pancreas and adrenal glands—are visualized routinely using CT. Unfortunately, even with high-resolution scanners, a CT image is almost always less precise than it appears. The image comprises a matrix of tiny picture elements, each of which represents the average tissue density of a three-dimensional “slice” of brain that may be up to 10 mm thick. This phenomenon, known as volume averaging, limits the resolution of all current neuroimaging technologies.

### Magnetic Resonance Imaging

**Figure 2:**
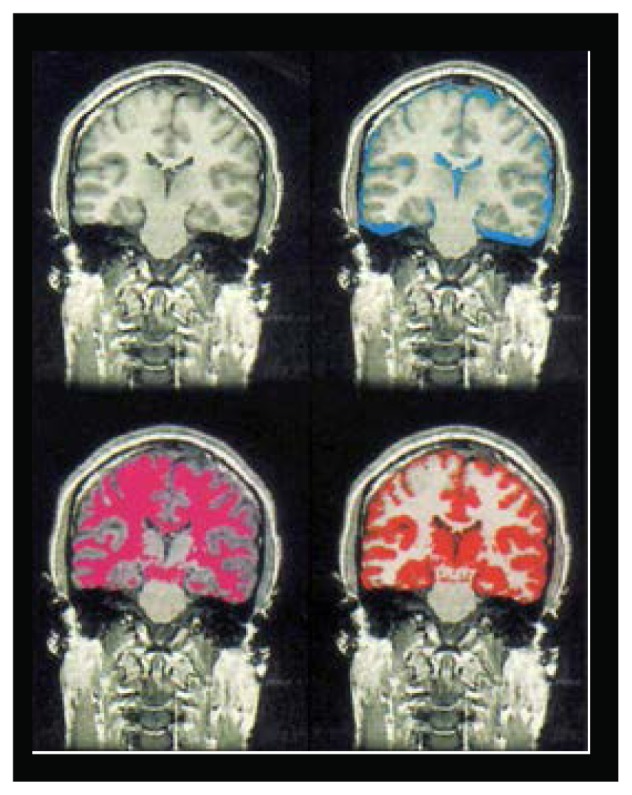
Magnetic resonance imaging can provide more detailed structural information about the brain than is obtainable using computed tomography. Image courtesy of Daniel Hommer.

Since the mid-1980’s, magnetic resonance imaging (MRI) has been replacing CT as the method of choice for many imaging applications. In MRI, the patient is placed in a chamber and exposed to radio waves in the presence of a powerful magnetic field. This treatment causes the nuclei of certain atoms in the brain to emit signals as they align and realign within the magnetic field. Different tissues of the body produce different signals, depending on the mixture of elements they contain. MRI scanners translate the signals into three-dimensional images that can delineate specific structures within the brain (figure 2).

Because hydrogen is the most abundant element in biological tissue, MRI scanners generally form images by detecting hydrogen nuclei. The signals that result arise predominantly from water and, to a smaller extent, from fatty substances (i.e., lipids). Tissues are thus differentiated based on the water content.

An MRI image is superior to that produced by X rays or CT scans; structures in the brain can be seen in fine detail, with clear differentiation among white and gray matter and CSF. Because MRI uses magnetism instead of X rays, a subject can undergo repeated MRI scans without the risk of exposure to potentially harmful radiation. However, the high level of magnetism may be hazardous in subjects with intracranial surgical clips or pacemakers. In addition, some subjects experience claustrophobia within conventional test chambers and therefore cannot be examined.

## Functional Imaging

The techniques described above have been used to study brain *structure*. Recent advances in imaging technology permit scientists to observe brain *function* as well by tracking chemical and physical processes associated with nerve cell activity.

### Functional MRI

One way to assess brain function is by tracing blood flow to different regions of the brain. In general, blood flow increases in areas undergoing high metabolic activity and decreases in areas affected by illness or injury. Traditionally, cerebral blood flow has been measured by introducing a radioactive tracer, such as xenon-133, into the body either by injection or inhalation. Radiation sensors placed over the surface of the scalp detect regional levels of radioactivity as blood flow distributes the xenon through the brain.

Refinements in MRI technique in the past few years have allowed researchers to gain important information about regional blood flow. These studies are generally performed with very rapid imaging sequences that are capable of obtaining many slices in just a few seconds. This method has shown promise in localizing normal motor, sensory, visual, and auditory functions and has been used to map cortical areas in patients before surgery. With the increasing availability of ultrafast MRI machines, this technique is expected to grow rapidly in the next several years.

### Magnetic Resonance Spectroscopy

**Figure 3:**
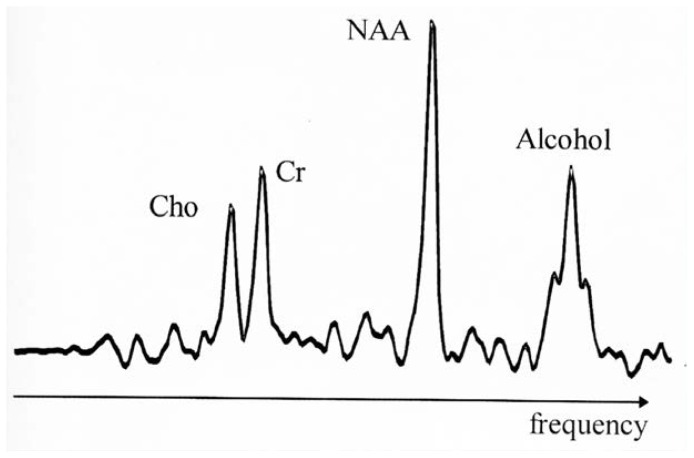
This magnetic resonance spectrum demonstrates the presence in a human brain of naturally occurring choline-containing compounds (Cho), creatinine-containing compounds (Cr), and *N*-acetylaspartate (NAA). The fourth peak at the right side of the spectrum indicates that the subject consumed alcohol before testing. Figure courtesy of George Fein.

Magnetic resonance spectroscopy (MRS) applies the principles of MRI to the examination of certain aspects of brain function. Conventional MRI detects hydrogen. MRS uses the MRI scanner to detect molecules such as lipids, amino acids (the building blocks of proteins), and certain substances within nerve cells that are involved in energy production (figure 3). The resulting spectra and color-coded images provide detailed information about metabolic activity in different parts of the brain.

Unlike many functional imaging techniques, MRS does not expose the subject to radioactivity. Thus MRS, like MRI, can be used repeatedly on the same subject, allowing researchers to track metabolic changes over time. MRS also can be used to study the function of the liver, heart, and kidney.

### Single-Photon Emission Computed Tomography

Single-photon emission computed tomography (SPECT) combines the use of radioactive tracers with the computer technology used in CT. In SPECT, cameras circle the head of a supine or seated subject, detecting radioactivity emitted from tracers such as iodine-123 and technetium-99m. A computer can display the resulting data in the form of sequential color-coded tomographic sections. Spatial resolution of SPECT is not as precise as x-ray CT, but variations in blood flow can be detected in areas on the order of 1 cm resolution.

**Figure 4:**
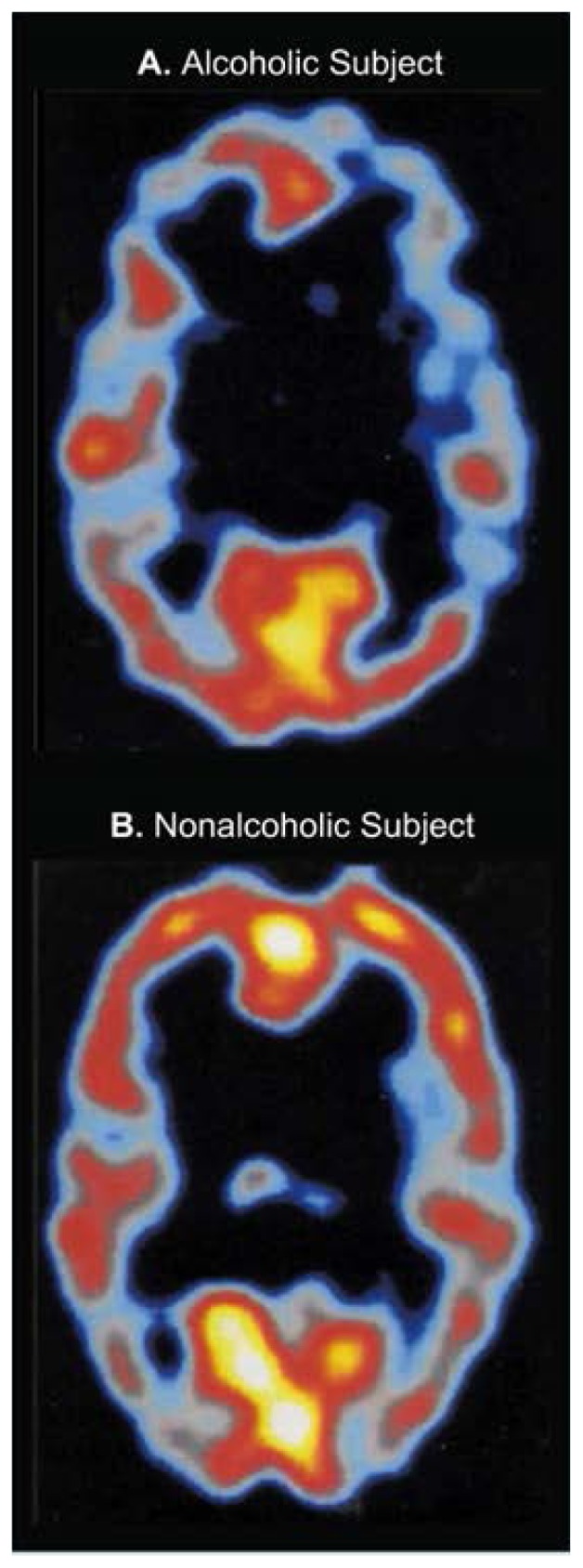
Some effects of alcohol may be related to interactions with the chemical messenger gamma-aminobutyric acid (GABA) in the brain. Results of single-photon emission tomography suggest differences in GABA function between (A) a 26-year-old alcoholic man and (B) a 26-year-old nonalcoholic man. These images were obtained using a radioactive tracer that binds to brain proteins involved in GABA function. Image courtesy of Anissa Abi-Dargham.

Most SPECT studies as currently performed reflect a combination of both cerebral blood flow and metabolism, because most clinically used isotopes are distributed according to blood flow and not taken up according to regional metabolism. In addition, special tracers have been developed for observing the activity of the brain’s neurotransmitters, chemicals responsible for communication between nerve cells. Studies using these tracers may help researchers determine how alcohol produces its effects on the nervous system (figure 4).

### Positron Emission Tomography

**Figure 5:**
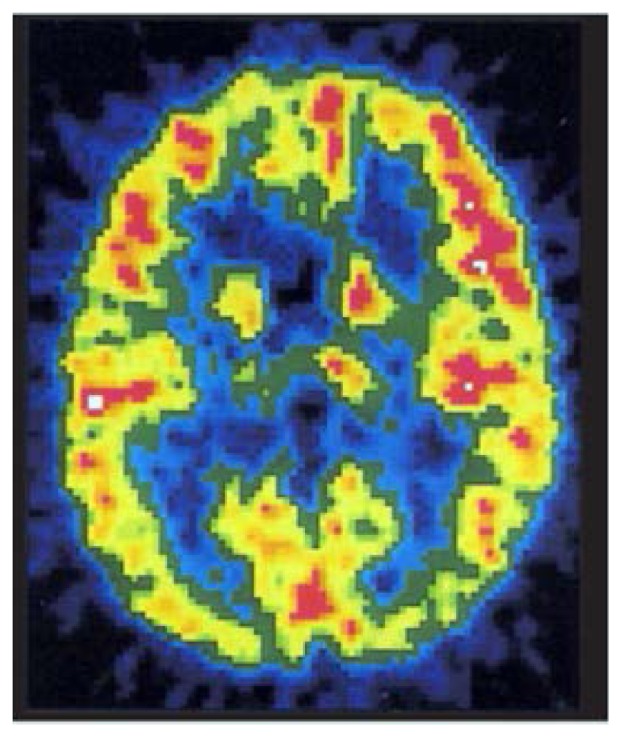
Positron emission tomography, the most powerful of current imaging tools, uses radioactive tracers to study brain energy metabolism and blood flow. Image courtesy of Daniel Hommer.

Positron emission tomography (PET) is the most powerful, and also the most costly, of current functional imaging tools (figure 5). PET employs tracers that are radioactive variants (i.e., radioisotopes) of elements found naturally in biological tissue. These tracers can be chemically attached to compounds involved in normal brain metabolism and injected into the bloodstream.

The brain function indicators that have been studied most widely with PET are energy metabolism and blood flow. Energy metabolism can be measured using glucose^2^—normally the brain’s sole energy source—labeled with the tracers fluorine-18 or carbon-11. Cerebral blood flow can be measured in various ways, including with water labeled with oxygen-15.

Using specially prepared tracers, researchers can investigate aspects of nerve cell communication, as with SPECT. The location of PET tracers in the brain is determined by radioactivity detectors arranged in a ring around the subject’s head. A computer then integrates the resulting series of tomographic images into a three-dimensional image of the brain that shows the distribution of the tracer-labeled compound. Current PET scanners can locate a source of radiation to within 5 to 10 mm of surrounding brain tissue.

Many of the isotopes employed in PET are short-lived, with a half-life of as little as 2 minutes. Because the radioactivity dose the subject receives is relatively low, PET can be used for sequential studies in the same subject to follow the course of a disease; it can also be used with relative safety to study healthy people.

The rapid decay of many PET tracers is also a disadvantage, however. Most radioisotopes needed for PET are produced in a cyclotron. The cyclotron must be located close to the subject so that such isotopes can be injected into the subject as soon as they have been produced. Thus, only institutions that have close access to a cyclotron can perform PET scanning with the full range of isotopes available. Production of all these isotopes is labor intensive and expensive. Less expensive generators and kits are available for other commonly employed PET isotopes.

### Electroencephalography

**Figure 6:**
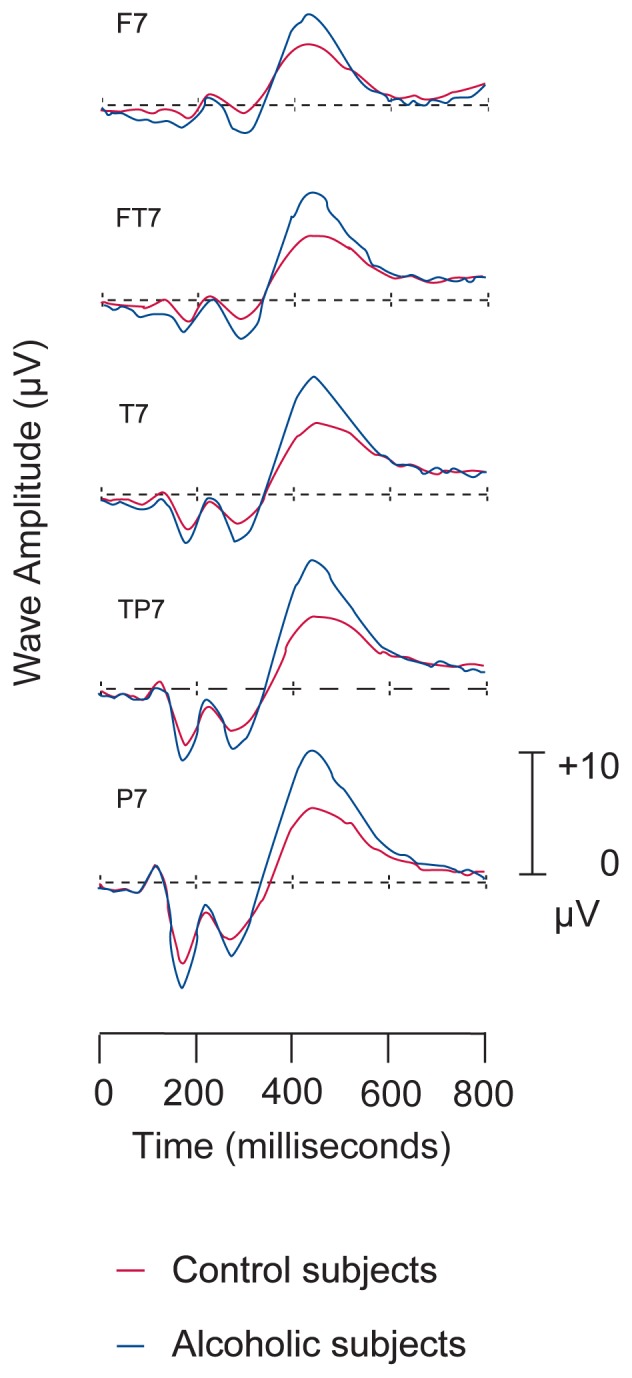
Electroencephalographic (i.e., brain wave) tracings of the wave form P300 obtained from nonalco-holic (i.e., control) and alcoholic subjects in response to a visual stimulus. Each tracing represents a different location on the scalp. The vertical scales represent wave amplitude measured in microvolts (μV) of electrical potential. Figure courtesy of Bernice Porjesz.

The electroencephalogram (EEG) records the spontaneous electrical activity of the brain created by nerve cell activity, or firing. Electrical signals from the brain are detected by means of electrodes placed in standard positions on the scalp. Characteristic brain wave patterns are associated with different mental and behavioral states. These patterns can be analyzed qualitatively or by computer to reveal the effects of drugs, metabolic conditions, and injury on the electrical aspects of brain function. Alcohol researchers commonly employ a variation of an EEG technique that measures event-related potentials (ERP’s), specific electrical responses to sensory stimuli, such as sights or sounds (figure 6). Brain electrical responses can be detected within a fraction of a second following the presentation of a stimulus. Using ERP’s, researchers can evaluate the efficiency of nearly instantaneous aspects of information processing, such as attention and recognition.

### Magnetoencephalography

**Figure 7:**
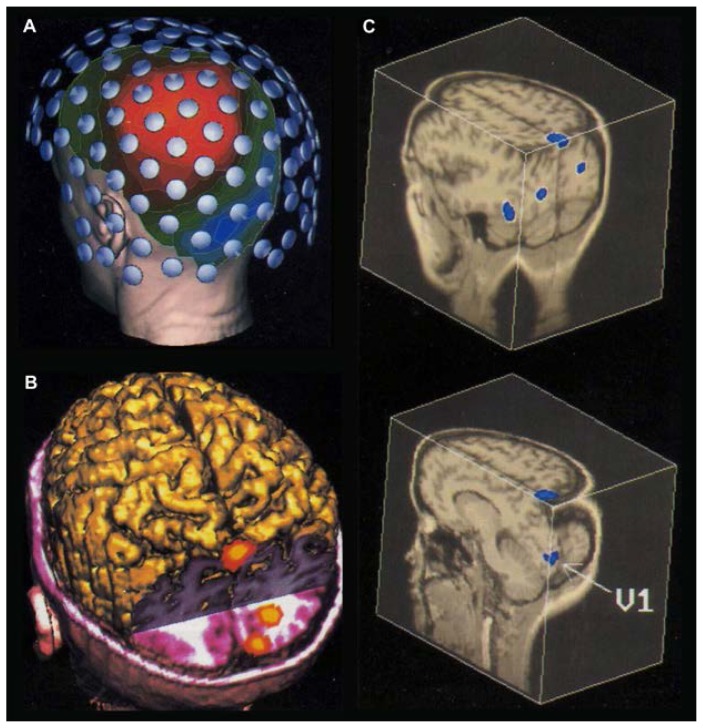
(A) Magnetoencephalogram evoked by a small visual stimulus in the subject’s lower right visual field. Magnetic field distributions (blue-red contours) are superimposed on a magnetic resonance-derived image of the subject’s head. Magnetic fields are displayed in red as they emerge from the head and in blue as they reenter. The white dots represent locations of the magnetic sensors. The data represent an average of 100 individual responses. (B and C) Vision-related areas of the brain’s outer layer (i.e., the cortex) are displayed by spatiotemporal analysis of the brain’s magnetic response to visual stimuli. Magnetic field sources are displayed in B as yellow-red contours and in C as blue contours. In B and C, magnetic field sources are displayed on images of the subject’s cortex derived from magnetic resonance imaging. Images courtesy of Doug Ranken.

The same nerve cell activity that produces the EEG also produces a magnetic field surrounding the head. The measurement and display of this field constitutes a magnetoencephalogram (MEG) (figure 7). The magnetic detectors commonly used operate at an extremely low temperature within a container of liquid helium. This container is shaped to allow magnetic field measurements at about 1 cm from the subject’s scalp. Interference from background magnetism (e.g., from the earth’s magnetic field and any electrical machinery in the vicinity) is suppressed in part by conducting the MEG measurements in a magnetically shielded room.

Although the bones of the skull tend to hamper the transmission of electric fields, these bones are largely transparent to magnetic fields. Therefore, brain regions can be localized more easily and rapidly with MEG than with EEG. New methods using large arrays of detectors can pinpoint magnetic field patterns of brain activity to within 10 to 20 mm. Data can be displayed as wave forms, as with EEG. Because MEG measures nerve cell firing differently from EEG, combining the two techniques may improve the ability to accurately localize the effects of alcohol on the brain.

## Footnotes

1 Computed tomography is the imaging of thin slices of an organ.

2 In practice, researchers use a chemical variant of glucose.
